# Fatal Rhinocerebral Mucormycosis and *Aspergillus flavus* Coinfection Complicated by Internal Carotid Artery Arteritis: A Case Report

**DOI:** 10.1155/carm/9945781

**Published:** 2026-06-30

**Authors:** Mukhtar Ansari, Talal Alhumaidi Alshammari, Majed Mamdouh Alshammari, Yasser Alhabeeb, Nada Nasser Almansour, Razan Abdullah Alshammari, Alya Khaled Alshayia

**Affiliations:** ^1^ Department of Clinical Pharmacy, College of Pharmacy, University of Ha’il, Hail, Saudi Arabia, uoh.edu.sa; ^2^ Oral & Maxillofacial Surgery Department, King Khalid Hospital, Hail Health Cluster, Hail, Saudi Arabia, ksu.edu.sa; ^3^ Department of Clinical Pharmacy, King Khalid Hospital, Hail Health Cluster, Hail, Saudi Arabia, ksu.edu.sa; ^4^ Internship, College of Pharmacy, University of Ha’il, Hail, Saudi Arabia, uoh.edu.sa

**Keywords:** arteritis, *Aspergillus flavus*, cerebral infarction, coinfection, diabetes mellitus, internal carotid artery, mucormycosis

## Abstract

This case report describes a fatal case of rhinocerebral mucormycosis and *Aspergillus flavus* coinfection in a 63‐year‐old Saudi male with poorly controlled Type II diabetes and hypertension. The patient presented with a 2‐day history of rapid right‐sided facial swelling, blurred vision, and proptosis. Clinical examination revealed necrotic nasal mucosa and ophthalmoplegia, while laboratory tests showed critical hyperglycemia (RBS 500 mg/dL) and severe leukocytosis. CT and MRI imaging confirmed extensive invasive fungal sinusitis with spread into the right cavernous sinus and involvement of the internal carotid artery (ICA). This angioinvasive progression resulted in infectious ICA arteritis and multiple acute cerebral infarcts. Management involved high‐dose liposomal amphotericin B (escalated to 7.5 mg/kg), functional endoscopic sinus debridement, and initially voriconazole, which was discontinued due to hemodynamic instability. Despite these aggressive interventions and histopathological confirmation of the mixed fungal infection, the patient’s neurological status deteriorated, leading to mechanical ventilation and, ultimately, death. This case illustrates the lethal nature of rhinocerebral mucormycosis, particularly when complicated by vascular invasion and coinfection. In regions like Saudi Arabia, where diabetes prevalence is high, a high index of clinical suspicion and immediate metabolic and surgical intervention are vital, as prognosis remains exceptionally poor once the infection reaches the cavernous sinus.

## 1. Introduction

Mucormycosis is an uncommon but highly aggressive angioinvasive fungal infection caused by fungi of the order Mucorales and class Zygomycetes. It is characterized by rapid vascular invasion, tissue necrosis, and a fulminant course if not recognized and treated promptly [1, 2]. Mucormycosis represents a critical opportunistic infection that often results in prolonged health complications and mortality for patients that require urgent intervention [3]. The rhinocerebral form represents one of the most serious presentations, which frequently begins with nonspecific sinonasal symptoms and rapidly progresses to orbital and intracranial involvement. Once the disease extends to the cavernous sinus, internal carotid artery (ICA), or brain parenchyma, prognosis significantly worsens, with mortality rates frequently exceeding 50% despite aggressive therapy [1, 2, 4, 5].

Uncontrolled diabetes mellitus is the most common cause worldwide. Hyperglycemia impairs neutrophil chemotaxis and phagocytosis, while ketoacidosis increases availability of iron in the serum due to its dissociation from iron‐binding proteins under acidic conditions, creating a permissive environment for fungal proliferation [2, 3, 6]. Other risk factors include malignancies due to chemotherapy or immunotherapy, organ transplantation, exposure to corticosteroid, and prolonged neutropenia [7].

From a regional perspective, Saudi Arabia and neighboring Gulf countries have a particularly high onset of mucormycosis due to the elevated prevalence of diabetes mellitus. Nearly half of the 33 confirmed cases in a multicenter retrospective study from Saudi Arabia (2009–2019) had diabetes, and 18% had rhinocerebral involvement. Despite antifungal and surgical therapy, mortality rate reached 48% [8]. Rhino‐orbital‐cerebral mucormycosis represented 26.7% of the 15 cases in another group from the western region, and the mortality rate was 73.3% [9]. Furthermore, the most recorded form of mucormycosis is rhinomaxillary mucormycosis [10].

The development of mixed fungal infections, such as concomitant Mucorales and *Aspergillus* species, has further complicated management, which requires early initiation of liposomal amphotericin B, aggressive surgical debridement, and strict metabolic optimization [1, 4, 6]. Early surgical excision of the infected tissue and repeated surgical debridement and exploration can often prevent the infection from spreading to the eye in rhinocerebral mucormycosis, thereby eliminating the need for enucleation and improving the prognosis [2].

## 2. Case Report

### 2.1. Patient Information

The patient was a 63‐year‐old Saudi male with a history of poorly controlled Type II diabetes mellitus and hypertension. His family history was noncontributory, with no reported instances of similar clinical presentations, hereditary disorders, or chronic medical conditions.

### 2.2. Clinical Findings

The patient presented with a 2‐day history of rapidly progressive symptoms including the following:

• Right‐sided facial swelling and cellulitis extending to the hard and soft palate.
• Blurred vision, retro‐orbital pain, subjective fever, and loss of balance.
• Physical examination revealed proptosis, eyelid edema, a fixed dilated pupil, and complete ophthalmoplegia.
• Additional findings included an absent corneal reflex, necrotic nasal mucosa, and necrosis in medial canthal tissue.

### 2.3. Timeline (Table 1)

• Day 0–2: Rapid onset of facial swelling and visual disturbances were observed.
• Admission: Clinical examination and laboratory tests were performed, and aggressive antifungal and antibiotic therapy was initiated.
• Diagnostic phase: CT and MRI/MRA imaging confirmed extensive fungal invasion and arterial involvement.
• Intervention: Functional endoscopic sinus debridement and biopsy were conducted.
• Progression: Despite receiving the most treatment possible, neurological deterioration resulted in the need for mechanical ventilation.
• Outcome: The patient passed away following extensive cerebral infarction and systemic complications.

**TABLE 1:** Timeline about chronology of the disease and its progression.

Timeline	Clinical event and interventions
Days 1–2	Rapid development of right‐sided facial swelling, blurred vision, retro‐orbital pain, and loss of balance.
Admission	Presentation with proptosis, ophthalmoplegia, and necrotic nasal mucosa. Laboratory tests confirmed critical hyperglycemia and severe leukocytosis.
Diagnostic phase	CT and MRI confirmed invasive fungal sinusitis, cavernous sinus invasion, and infectious internal carotid artery (ICA) arteritis.
Initial therapy	Started on liposomal amphotericin B (5 mg/kg) and voriconazole. Voriconazole later discontinued due to hemodynamic instability and atrial fibrillation.
Surgical phase	Functional endoscopic sinus debridement was performed. Histopathology and cultures confirmed Mucormycosis and *Aspergillus flavus*.
Escalation	The dose of amphotericin B was escalated to 7.5 mg/kg due to the aggressive nature of the infection.
Deterioration	Development of multiple acute cerebral infarcts in the right hemisphere and left‐sided weakness. Patient required mechanical ventilation.
Final outcome	Despite aggressive medical and surgical management, the patient died from the infection.

### 2.4. Diagnostic Assessment

#### 2.4.1. Laboratory Results

Laboratory investigations revealed marked leukocytosis (WBC 34 × 10^9^/L) indicating severe infection, critical hyperglycemia (RBS 500 mg/dL), acute inflammation indicated by C‐reactive protein (CRP 210 mg\L), and erythrocyte sedimentation rate (ESR 145 MM/HR) (see Table 2). The patient’s poorly controlled Type II diabetes provided the classic permissive environment for Mucorales. The main clinical indicator of an angioinvasive fungal process was the existence of necrotic nasal mucosa and medial canthal tissue.

**TABLE 2:** Laboratory profile in rhinocerebral mucormycosis and *Aspergillus flavus* coinfection: diagnostic findings and treatment monitoring parameters.

Laboratory tests	Result	Reference range	Interpretation
WBC	34 × 10^9^/L	4.0–11.0 × 10^9^/L	Severe infections/immune response
RBS	500 mg/dL	80–180 mg/dL	Critical hyperglycemia
CRP	210 mg\L	< 5–10 mg/L	Acute systemic inflammation
ESR	145 MM/HR	20 mm/h	Extreme chronic or acute inflammation

#### 2.4.2. Imaging

##### 2.4.2.1. Most Important Findings

**FIGURE 1:**
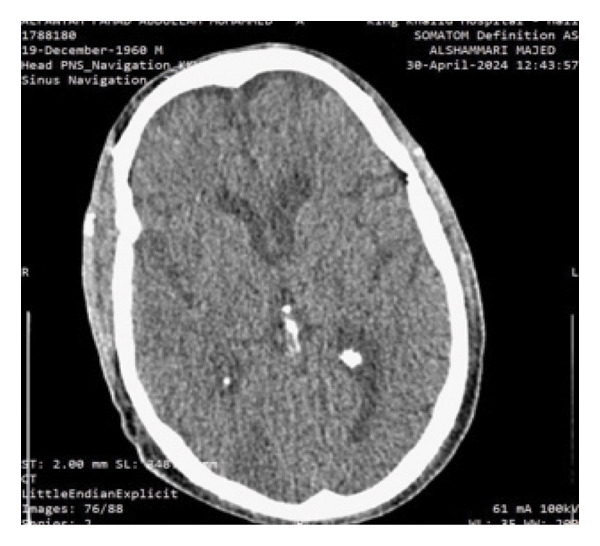
Axial MRI/CT showing cavernous sinus invasion and multiple acute cerebral infarcts in the right hemisphere.

Cavernous sinus invasion: There was no enhancement at all because the invasive tissue had entered the right cavernous sinus. This suggests thrombosis or invasion of the right cavernous sinus (MRI/MRA: Figure 1).

ICA involvement and arteritis: The right ICA’s cervical, petrous, and cavernous segments had minor attenuation, a high T2 signal, and diffuse mild wall thickening. These results strongly suggest severe, angioinvasive viral ICA arteritis, even if the artery is still patent (not completely clogged) (MRI/MRA: Figure 1).

Acute cerebral infarcts: The patient had experienced several acute right cerebral infarcts (strokes) as a result of the vascular invasion and arteritis (MRI/MRA: Figure 1).

##### 2.4.2.2. Secondary Findings

Soft tissue spread and sinus necrosis: Nonenhancing, necrotic tissue and extensive right‐sided sinusitis (maxillary, ethmoid, and sphenoid) were seen in CT and MRI. The MRI further shows that the infection had spread to the pterygopalatine fossa, nasopharyngeal/right parapharyngeal spaces, periantral region, infratemporal fossa, and right nasal cavity.

**FIGURE 2:**
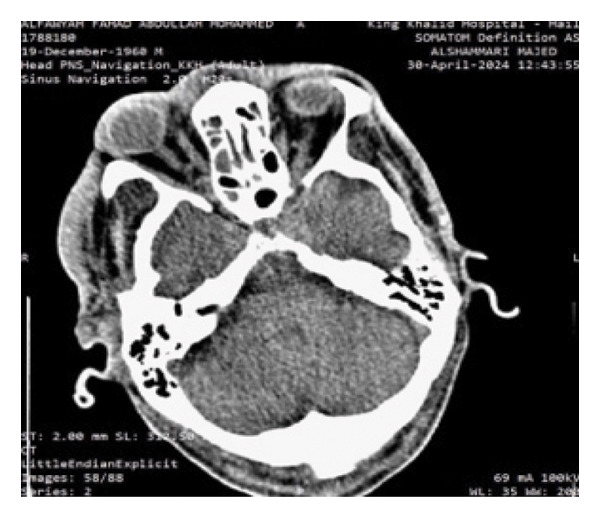
Axial CT scan showing right‐sided sinusitis with bony erosion of the anterior and superior orbital walls.

**FIGURE 3:**
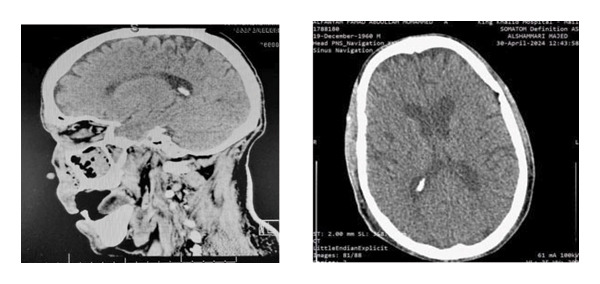
Sagittal CT scan demonstrating extensive opacification of the maxillary antrum and ethmoidal sinusitis.

Orbital invasion: There was considerable bony erosion of the lamina papyracea and superior wall (orbital floor), which spreads infection into the orbital cavity. This has resulted in proptosis, inferior rectus muscle displacement, and a slight subperiosteal collection (CT scan: Figures 2 and 3). Although the intraconal orbital fat was still visible, the right orbital extraconal and preseptal spaces were extended as seen in MRI.

#### 2.4.3. Histopathology and Microbiology

Mucormycosis was supported by both histopathological findings and fungal culture, while PCR testing was not performed.

• Incisional biopsy: Histopathological findings of necrotic nasal tissue demonstrated broad nonseptate hyphae with right‐angle branching and angioinvasion which confirmed mucormycosis.
• Fungal culture: Cultures grew *Aspergillus flavus*, confirming a mixed (Zygomycetes/Mucorales) fungal coinfection.
• Differential diagnosis. A fatal coinfection of rhinocerebral mucormycosis and *Aspergillus flavus* was diagnosed through imaging and histopathological confirmation.

### 2.5. Therapeutic Interventions

The patient was treated aggressively using multiple modalities:

• Pharmacotherapy: The patient was initially started on intravenous Augmentin and metronidazole and subsequently escalated to meropenem and vancomycin for presumptive meningitis.
• Antifungal therapy: High‐dose of liposomal amphotericin B (escalated from 5 to 7.5 mg/kg) was administered. Additionally, voriconazole was initially administered but discontinued due to atrial fibrillation and hemodynamic instability.
• Surgery: The patient underwent functional endoscopic sinus debridement.

### 2.6. Follow‐Up and Outcomes

Despite aggressive medical and surgical interventions, the patient’s condition worsened:

• He developed multiple acute cerebral infarcts in the right ACA/MCA watershed territory.
• Neurological deficits progressed to left‐sided weakness and required intubation and mechanical ventilation.
• Plans for right eye enucleation were made, but the patient passed away before further surgical intervention could be completed.

## 3. Discussion

Rhinocerebral mucormycosis is a fulminant and often fatal opportunistic fungal infection that demands immediate clinical consideration. This case highlights the aggressive nature of the disease, particularly when complicated by a coinfection with *Aspergillus flavus*.

Uncontrolled diabetes mellitus remains the most significant risk factor for mucormycosis. The critical hyperglycemia (RBS 500 mg/dL) and subsequent metabolic instability created a permissive environment for fungal proliferation in this patient [3]. Hyperglycemia is known to impair essential immune functions, such as neutrophil chemotaxis and phagocytosis. Furthermore, if ketoacidosis is present, the acidic environment causes iron to dissociate from binding proteins, providing the fungi with the iron necessary for rapid growth [11, 12]. This patient’s laboratory profile—characterized by severe leukocytosis (WBC 34 × 10^9^/L) and extreme inflammatory markers (CRP 210 mg/L; ESR 145 mm/hr)—reflects a massive systemic inflammatory response to this invasive process [13].

A hallmark of mucormycosis is its angioinvasive nature. The fungus penetrates blood vessels, leading to thrombosis and subsequent tissue necrosis. In this case, MRI and MRA findings show that the infection had progressed to involve the cavernous sinus and the ICA. The development of infectious ICA arteritis is a fatal complication of rhinocerebral mucormycosis. Although the vessel remained patent, the resulting vascular inflammation likely contributed to the multiple acute cerebral infarcts observed in the right ACA/MCA territories and diffuse wall thickening due to fungal angioinvasion. This case shows that although some cases have complete vascular occlusion, even nonocclusive arteritis can result in deadly neurological degeneration once the infection reaches the cavernous sinus. While mortality rate from mucormycosis is generally over 50%, the disseminated form is extremely deadly, with deaths exceeding 90%. Survival is rare at that stage unless medical intervention is given immediately [14].

Similar to this case, several documented cases of mixed fungal infections highlight the need for urgent surgical intervention and early metabolic optimization. The presence of *Aspergillus flavus* can complicate the pharmacological profile even though mucormycosis is characterized by fast tissue necrosis and a fulminant course. This was evident when the patient’s hemodynamic instability required stopping voriconazole, a typical treatment for aspergillosis [15].

The aggressive nature of rhinocerebral mucormycosis is well documented, but its heterogeneous etiology and severe vascular complications make this case more complicated. The fact that mixed infections including both *Aspergillus* and Mucorales species provide a clinical challenge that calls for integrated therapy strategies such as liposomal amphotericin B and surgical debridement is becoming increasingly recognized. Although rhinocerebral involvement is common in diabetic individuals, regional data from Saudi Arabia show that mortality is still high, ranging from 48% to as high as 73.3% in rhino‐orbital‐cerebral presentations. While liposomal amphotericin B and surgical debridement are the gold standards of treatment, the rapid progression of neurological deficits in this case highlights that even aggressive therapy may not be sufficient once intracranial extension and vascular compromise have occurred [3, 16, 17].

### 3.1. Strengths and Limitations

The case provides a conclusive diagnosis through a combination of modern neuroimaging (CT, MRI, and MRA) and gold‐standard confirmation through both histopathology and fungal cultures. Additionally, it provides a thorough description of aggressive multidisciplinary care, including surgical debridement and the titration of high‐dose liposomal amphotericin B to 7.5 mg/kg.

Limitations of the case include the single‐case nature, inability to explore alternative antifungal strategies in depth due to rapid deterioration, and a lack of long‐term follow‐up. The primary limitation was the patient’s inability to tolerate dual antifungal therapy due to hemodynamic instability and atrial fibrillation. Furthermore, the window for effective intervention was severely limited due to the infection’s advanced stage upon presentation, which already involved the ICA and cavernous sinus.

## 4. Conclusion

This case highlights the lethal progression of rhinocerebral mucormycosis in a patient with poorly controlled diabetes mellitus. The rapid transition from sinonasal symptoms to infectious carotid arteritis and cerebral infarction demonstrates the devastating speed of fungal angioinvasion. Despite aggressive management with high‐dose liposomal amphotericin B and surgical debridement, the prognosis remains exceptionally poor once the infection reaches the cavernous sinus. In regions with high diabetes prevalence like Saudi Arabia, maintaining a high index of suspicion is vital for early diagnosis. Early diagnosis, strict glycemic control, and immediate surgical and antifungal intervention are the only ways for potentially improving survival outcomes in this highly aggressive disease.

## Funding

No funding was obtained for this study.

## Ethics Statement

This case study received ethical approval from Research Ethics Committee, Hail Health Cluster, Hail, Saudi Arabia.

## Consent

The patient’s legal guardian provided written informed consent for the publication of this case report and related clinical photos. For ethical reasons, the patient’s identity has been kept anonymous.

## Conflicts of Interest

The authors declare no conflicts of interest.

## Data Availability

All information pertinent to this case, including clinical findings and laboratory results, is documented within the case.
